# Stricture of the Œsophagus, from Swallowing Sulphuric Acid, Successfully Treated

**Published:** 1827-10

**Authors:** W. Taylor

**Affiliations:** Surgeon.


### STRICTURE OF THE ŒSOPHAGUS.

*Stricture of the Œsophagus, from swallowing Sulphuric Acid, successfully treated.* By W. Taylor, Surgeon.

Master H—, ætatis twelve, a fine healthy boy, had the misfortune, on the 19th of December, 1826, to swallow by mistake a quantity of highly concentrated sulphuric acid. When the accident occurred, he was a pupil at Mr. Stock's academy at Poplar. Mr. Tatum, a practitioner residing in the immediate vicinity of Mr. Stock's residence, was requested to attend immediately: Mr. T. very promptly obeyed the summons, and the usual antidotes recommended in such cases were quickly administered,—viz. magnesia, with small quantities of carbonate of soda, in mucilaginous drinks, and I believe emetics. Eight ounces of blood were taken from the arm the same evening. Mr. Tatum was of opinion that a quantity of acid had passed into the stomach; but I am inclined to be sceptical upon this point, as the boy never complained of any pain about the epigastric region.

Dec. 20th, (the day after the accident,) he was removed to his father's residence, and I was requested to see him. He complained of excessive heat and soreness in his mouth and throat, with great thirst. His pulse was frequent, but not full.

I prescribed two drachms of Castor-oil, blended with mucilage, every four hours, till his bowels were relieved; and directed his attendants to give him some warm milk and water frequently.

21st.—I visited him again at eight o'clock A.M. The castor oil had not produced any evacuation from the bowels; he had passed a tolerably good night, having had about four hours' sleep, though occasionally starting up in great alarm.

I ordered a clyster of mutton-broth and gruel to be administered every two hours; and the dose of Castor-oil to be augmented, and repeated more frequently.

Six o'clock P.M.—His bowels had been freely evacuated. The pulse was full and frequent; the skin hot, and thirst urgent.

Ten ounces of blood were taken from the arm; desired his attendants not to give him any thing but warm milk.

22d.—Had passed a good night; his bowels had been freely opened; pulse tranquil; skin cool, and thirst diminished.

23d.—The cuticle of the tongue and fauces separated; quite free from pain; ate his bread and milk night and morning, and a little bread-pudding for his dinner, with perfect facility.

From this period till the 8th of January, he appeared to improve daily: in fact, he was apparently quite recovered. I was then requested to see him, as he had experienced great difficulty in swallowing, accompanied with sickness. Finding his tongue coated, and bowels confined, I attributed the interruption of his recovery to a disturbed state of the stomach, as he had recently been indulging in the excesses so frequent at that season.

I prescribed an aperient powder to be taken occasionally, containing one grain of Calomel, three of Rhubarb, and one of Ginger; also a grain of Sulphate of Quinine three times a-day; and ordered him a light nutritive diet.

This plan of treatment was strictly pursued, without any very manifest amendment of his complaint, though his general health and spirits appeared much benefited by it, till the 18th of January, when I considered it advisable to have another opinion.

Mr. ABERNETHY was consulted, who thought it a spasm of the

œsophagus, with derangement of the stomach, and recommended the aperient powder to be given every alternate night, the decoction of Sarsaparilla three times a-day, and the greatest possible attention to be paid to his diet. The plan of treatment suggested by Mr. Abernethy was adopted, but I cannot say faithfully: although the utmost care was taken to prevent him from having access to food of an improper description, and at irregular periods, yet he found means to obtain it, and his sufferings were observed invariably to be aggravated by the indulgence. A lump of sugar, or a small piece of sweet cake, would occasion such a violent spasm of the œsophagus as to prevent him from swallowing his saliva for five or six hours.

January 26th.—For three or four days preceding this date, his friends thought him infinitely worse: his countenance was pallid, with obviously increasing debility, and great emaciation. The spasmodic contraction of the œsophagus was occasionally so extremely violent as to preclude the possibility of swallowing any solid, and but little liquid, food for many hours. Sir Astley Cooper was then consulted, who was of opinion that there was an ulcer in the œsophagus, as small portions of membrane were occasionally brought up. A grain of Sulphate of Quinine was prescribed for him three times a-day, with a mild aperient when requisite; and a most nutritive liquid diet was recommended; egg, milk, and sherry with sugar and nutmeg, were given frequently. He swallowed these very well, and his health, in the course of five or six days, had very manifestly improved.

February 9th.—Called upon Sir Astley Cooper again, although, as I have before observed, his general health had greatly improved since our last consultation: he still experienced extreme difficulty in swallowing even very small portions of solid, and occasionally liquid, food.

**Prescribed small doses of Oxymuriate of Mercury in Tincture of Bark.**

14th.—No amendment. The bougie was had recourse to, but it met with such resistance that Sir Astley deemed it prudent to discontinue its use for some time, till the spasmodic action of the œsophagus subsided.

19th.—No amendment. Prescribed ten drops of the Muriated Tincture of Iron three times a-day. From this period till the 2d of April, this mode of treatment was strictly followed, without any alleviation of his sufferings. Bougies of divers sizes, both elastic gum and metallic, were tried, but none would pass the stricture. Sir A. Cooper at length suggested quicksilver, and half a pound was administered twice a day for fifteen or sixteen days, without any very decided benefit: it was thought, occasionally during its use, that he had swallowed a little solid food with more facility; but the result was not attended with the degree of benefit that was anticipated: its use was consequently abandoned, as it produced pain and uneasiness in the bowels.

April 23d.—From this period till the 20th of May, bougies were regularly introduced every other day, without any manifest benefit; and, indeed, I have no hesitation in saying that they were most decidedly injurious. At length the mucous membrane of the œsophagus was slightly abraded, in consequence of the frequent and rather violent introduction of the instrument. This unfortunate occurrence brought on inflammation of the œsophagus, which precluded the possibility of his taking any food for fourteen or fifteen days. Extreme debility and great emaciation quickly followed, so that we were obliged to administer the most nutritive clysters twice or thrice a-day for five or six weeks. His health and spirits appeared gradually to improve; at length he was enabled to swallow bread and milk tolerably well, and in the course of a few days we discontinued the clysters.

He was sent into the country, and in a few weeks appeared in perfect health. He can now swallow a pint of bread and milk in half an hour for his breakfast, and three ounces of animal food at his dinner by careful mastication.

*42, Stamford-street, Blackfriars;*
*August* 1827.

